# Horizontal Ridge Augmentation with Xenogeneic Bone, Hyaluronic Acid, and Dermal Matrix by Tunnel Technique: A Case Series

**DOI:** 10.3390/dj14010025

**Published:** 2026-01-04

**Authors:** Giuseppe D’Albis, Marta Forte, Lorenzo Marini, Kezia Rachellea Mustakim, Andrea Pilloni, Massimo Corsalini, Saverio Capodiferro

**Affiliations:** 1 Department of Interdisciplinary Medicine, University of Bari “Aldo Moro”, Piazza Giulio Cesare, 11, 70124 Bari, Italy; massimo.corsalini@uniba.it (M.C.); saverio.capodiferro@uniba.it (S.C.); 2 Section of Periodontics, Department of Oral and Maxillofacial Sciences, Sapienza University of Rome, 00161 Rome, Italy; lorenzo.marini@uniroma1.it (L.M.); andrea.pilloni@uniroma1.it (A.P.); 3 Department of Oral and Maxillofacial Surgery, Hasanuddin University, Jl. Perintis Kemerdekaan No.KM.10, Tamalanrea Indah, Kec. Tamalanrea, Kota Makassar 90245, Sulawesi Selatan, Indonesia; keziarachelleamustakim@unhas.ac.id

**Keywords:** horizontal ridge augmentation, tunnel technique, xenogeneic bone graft, acellular dermal matrix, minimally invasive implant surgery, hyaluronic acid, dermal matrix

## Abstract

**Background**: Several minimally invasive techniques have been introduced to augment horizontal ridge volume for prosthetically driven implant placement, utilizing different biomaterials to enhance regenerative outcomes. This article presents two clinical cases illustrating a tunneling approach for horizontal alveolar ridge augmentation using a combination of xenogeneic bone graft, hyaluronic acid, and an acellular dermal matrix. **Methods**: A single vertical incision was made mesial to the bone defect and a dermal matrix was suitably shaped and positioned into the subperiosteal tunnel. Subsequently, the bone graft was inserted between the dermal matrix and the buccal bone plate. Primary wound closure was achieved. After six months, implants were placed. For each patient, an optical scan was performed at baseline (T_0_), at six months post-operative ridge augmentation surgery (T_1_) and at two months post-implant insertion (T_2_). A digital measurement of the horizontal ridge thickness was performed at each inserted implant site. Clinical parameters and patient postoperative morbidity were recorded. **Results**: The procedure was well tolerated by the patients. No postoperative clinical complications were observed. The mean tissue thickness achieved at T1 was recorded to be 13.3 mm. The same value was recorded at T2. **Conclusions**: This technique allowed the placement of prosthetically guided implants, with minimal morbidity and no observed complications. Further studies analyzing the histology of newly formed bone and performing three-dimensional radiological examinations to confirm the effectiveness of the surgical technique are warranted to validate these preliminary findings. Clinical Trial Number (NIH): NCT06424223

## 1. Introduction

The alveolar ridge augmentation is mandatory to insert implants in optimal prosthetic position when horizontal dimension is inadequate. To carry out the most successful regenerative surgery, a careful consideration of both technical and biological aspects must be fully considered when deciding on the appropriate technique [1,2]. Patient age, systemic disease comorbidities, oral hygiene maintenance, oral parafunction and compliance are certainly fundamental aspects to be evaluated before a regenerative surgery procedure. When treating patients with systemic diseases, clinicians should prioritize patient satisfaction and quality of life choosing a treatment with minimal risks and complications [3].

Several techniques have been introduced over time to minimize the invasiveness related to alveolar tissue augmentation [4] and mainly to prevent the coronal advancement of the flap and subsequent postoperative discomfort, swelling and complications. Among all, the generally called tunnel technique is surely a less invasive technique for the horizontal alveolar ridge augmentation as it involves making a vertical incision to access the bone defect and inserting the grafts, with the main benefit to avoid periosteal incisions. More precisely, different tunnel techniques have been described in the international literature using various biomaterials including bovine bone [5,6,7], synthetic bone [8], bovine bone block [9,10,11,12], allograft block bone [13,14], hidroxiapatite [15]. Generally, the use of membranes to prevent soft tissue invagination and stabilize bone graft is the most discussed aspect of the tunnel technique, although membrane management through a single incision may prove very complex and surely remains strictly operator dependent. In contrast, some authors suggested potential advantages by recombinant human platelet-derived growth factor-BB (rhPDGF-BB) alternatively to a protective graft membrane [16,17], but, at the present, such use is hindered in certain countries due to legal limitations, making it impossible to apply this technique worldwide.

A recent literature review comprehensively described all tunneling techniques applied in ridge augmentation procedures, highlighting their clinical protocols, materials used, and outcomes [18]. Across the reviewed studies, the elevation of the mucoperiosteal flap was consistently achieved using surgical instruments and periosteal elevators. Kfir et al. additionally described the employment of a balloon device, inflated within the surgical pocket, to facilitate completion of the tunneling procedure [19].

Several authors have reported performing decortication to harvest inductive bone cells from the marrow space, thereby enhancing osteogenic potential. Venet et al. utilized piezoelectric surgery for this phase to achieve greater precision and minimize trauma [13]. Among the techniques analyzed, the most variable aspect concerned the type of grafting material and its combinations. Studies reported the use of a membrane as a structural support for the graft. In these, collagen membranes were combined with autologous fibrin, synthetic bone, and bovine xenograft. Johnson et al. described the use of a dPTFE membrane in conjunction with an allogenic bone graft [10]. Other authors reported the placement of synthetic bone, rhPDGF-BB combined with bovine bone, allograft bone, xenograft bone, and stem cells associated with hydroxyapatite or autologous bone retrieved from the mandibular arch. To evaluate the volumetric outcomes, most studies employed two-dimensional measurements on CBCT scans obtained before the intervention and at 4–6 months post-operatively. Three studies also provided histological evidence of the quality of the regenerated tissue. Li et al. demonstrated that xenograft particles were embedded within woven bone nine months after bone block placement [9]. Moreover, new bone formation was histologically confirmed when rhPDGF-BB was used in combination with allogenic bone, mineralized collagen, and bovine bone.

The principal advantage of the tunnel approach in bone regeneration lies in its ability to avoid crestal incisions while preserving flap vascularization and minimizing trauma to the soft tissues. This characteristic is particularly beneficial for patients with vascular impairments, such as smokers, diabetics, or those presenting with fibrotic or scarred tissues. The lateral access also facilitates the placement of a substantial quantity of graft material through a minimal incision, allowing for tension-free primary wound closure. Tension-free closure markedly reduces the risk of membrane exposure or the need for postoperative antibiotic therapy, as typically required in open-flap techniques. No instances of suture dehiscence or early membrane exposure were reported in any of the included studies. Reduced postoperative morbidity and patient discomfort emerged as the most consistently reported clinical benefits. The successful application of these techniques requires adequate clinician training and careful selection of instruments and biomaterials. Particular caution must be exercised during the tunneling phase to prevent tissue perforation and preserve periosteal integrity. The absence of a direct view of the alveolar crest remains a potential limitation of the lateral approach in ridge augmentation [18].

This article presents two clinical cases treated with a tunnel approach and followed up for three years, employing a dermal matrix and bone chips mixed with hyaluronic acid for horizontal ridge augmentation. The patients included in this report are part of an ongoing clinical trial, and the complete data set will be published separately upon study completion.

## 2. Materials and Methods

This case series was conducted in accordance with the Declaration of Helsinki (1965), revised in Tokyo (2004). The study was registered under the identifier code NCT06424223 (Approval Date: 1 November 2022) at the National Center for Biotechnology Information of the National Library of Medicine (NIH). The study was approved by the Independent Ethics Committee of the Azienda Ospedaliero-Universitaria “Consorziale Policlinico” (Approval Code: 7390; Approval Date: 13 July 2022). After a thorough explanation of the protocol and the related risks and benefits, patients signed informed consent forms. Surgeries were performed by the same clinician (G.D.).

The surgical procedure was carried out under local anesthesia following standard aseptic protocols. The operative field was isolated, and a dermal substitute matrix (Mucoderm^®^, Straumann, Basel, Switzerland) was trimmed and pre-shaped to reproduce the ideal convex contour of the edentulous ridge. This adaptation was performed extraorally on a sterile surface to ensure an optimal three-dimensional fit within the recipient site.

**Figure 1:**
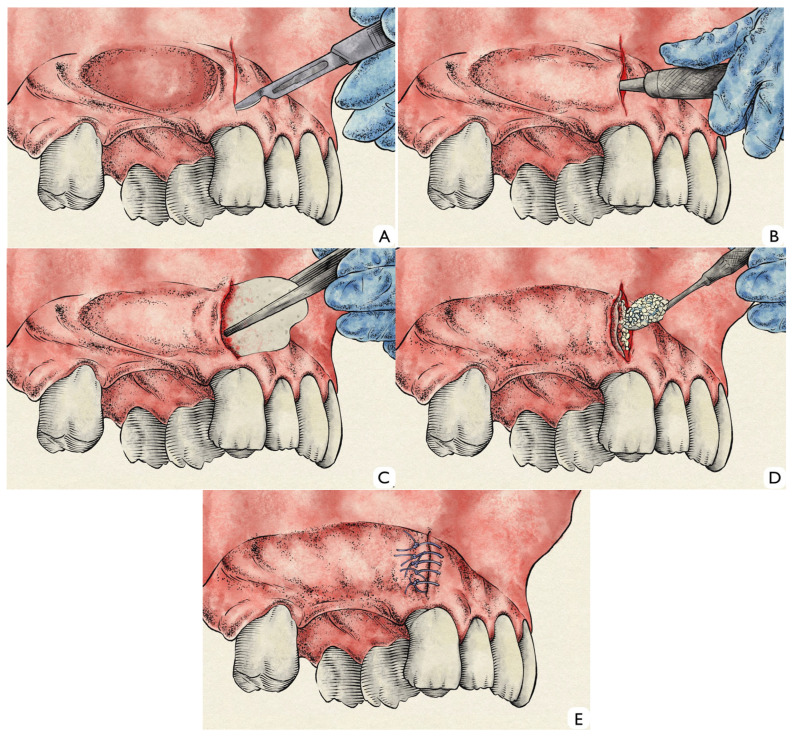
(**A**): Single vertical incision made mesial to the bone defect. (**B**): Flap detachment through lateral access. (**C**): Insertion of acellular dermal matrix into subperiosteal tunnel. (**D**): Placement of the bone graft between the dermal matrix and the cortical bone. (**E**): Primary intention suture of the flap.

A single vertical incision was made on the buccal alveolar mucosa, positioned approximately 1 cm mesial to the alveolar ridge defect and 4 mm apical to the gingival margin (Figure 1A). Through this access, a subperiosteal tunnel was carefully developed on the buccal aspect using fine periosteal elevators (Figure 1B). The tunnel dissection was extended sufficiently to allow the accommodation of the grafting materials, while taking great care to avoid flap perforations or tears, thus preserving the integrity and vascular supply of the periosteum. Once adequate tunnel volume was achieved, the dermal matrix was inserted subperiosteally (Figure 1C). The matrix was positioned without pre-hydration, intentionally maintaining its rigidity to facilitate handling and precise placement beneath the mucoperiosteal flap. A grafting mixture was then prepared by combining deproteinized bovine bone granules particle size range: 0.25–1 mm (Bio-Oss^®^, Geistlich, Wolhusen, Switzerland) with hyaluronic acid gel (xHyA^®^, Regedent, Zürich, Switzerland). The components were gently blended until a dense, cohesive compound was obtained, ensuring homogeneous distribution and preventing particle dispersion. This “sticky” composite graft was subsequently inserted into the tunnel and positioned between the internal surface of the dermal matrix and the external buccal bone plate (Figure 1D). The dermal matrix was not mechanically fixed to the surrounding tissues or bone. Wound closure was achieved by primary intention, using simple interrupted resorbable sutures (5-0 polyglactin; Vicryl^®^, Ethicon LLC, Somerville, NJ, USA) to approximate the vertical incision margins without tension (Figure 1E). Hemostasis was verified, and postoperative care included routine recommendations for oral hygiene, soft diet, and antimicrobial mouth rinses.

Postoperative management followed a standardized protocol designed to minimize infection, pain, and swelling while promoting optimal soft tissue healing. Following surgery, all patients were prescribed a broad-spectrum antibiotic regimen consisting of amoxicillin/clavulanic acid (875 mg/125 mg) administered orally twice daily for six consecutive days. The antibiotic course aimed to reduce the risk of postoperative infection associated with graft placement and soft tissue manipulation.

For pain control, ibuprofen 400 mg was prescribed to be taken as needed, with instructions not to exceed the maximum recommended daily dosage. Patients were advised to take the first dose approximately two hours after surgery to preempt the onset of postoperative discomfort.

To minimize postoperative edema and ecchymosis, patients were instructed to apply an ice pack intermittently over the operated area for the first 4 h, alternating 15 min applications with equal rest intervals. This approach was emphasized as particularly important during the immediate postoperative period to limit inflammatory swelling.

Oral hygiene instructions included the use of a 0.2% chlorhexidine mouth rinse, applied gently twice daily for two weeks to maintain antisepsis in the surgical field. Patients were advised to avoid mechanical trauma to the surgical site, including toothbrushing in the treated area, vigorous rinsing, or any excessive lip or cheek traction for at least four weeks. Dietary recommendations included a soft, non-chewy diet and avoidance of hot or spicy foods for the first 7–10 days. Smoking and alcohol consumption were strictly discouraged during the healing phase.

Postoperative evaluations were scheduled at 7 to 10 days for suture removal, during which tissue healing, inflammation, and mucosal integrity were clinically assessed. Any residual edema or erythema was documented. Subsequent follow-up visits were performed at 1 month, 3 months, and 6 months post-surgery to monitor soft tissue stability and assess patient comfort.

At the 6-month follow-up, a cone-beam computed tomography (CBCT) scan was obtained to evaluate the dimensional stability of the augmented ridge, bone volume gain, and integration of the grafting materials. Radiographic measurements were compared to baseline CBCT data to quantify horizontal ridge augmentation outcomes. A diagnostic wax-up of the missing teeth was performed on the digital casts. Each case was designed using a dental implant planning software (coDiagnostiX, version 10.5, DentalWings) to plan the implants position. The implant site was prepared after raising a full-thickness flap and the implants were placed as designed in digital planning with the support of a surgical template. The surgical procedures were performed in guided surgery according to the protocols recommended by implants manufacturers. The patients were recalled two months after implant surgery for a pre-prosthetic evaluation, the healing abutments were placed, and implant stability proven. Two weeks later, the definitive impression was digitally taken, and the following prosthetic procedures performed.

This study has been reported in line with the TREND guideline [20].

### 2.1. Case 1

**Figure 2:**
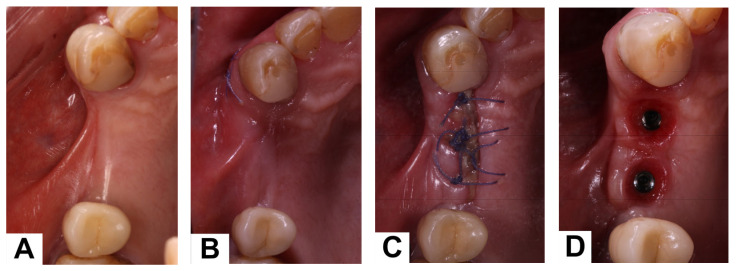
(**A**): Occlusal view of the initial situation. (**B**): Horizontal tissue augmentation after surgery. (**C**): Clinical situation after implant placement. (**D**): Peri-implant tissues before prosthetic delivery.

A 75-year-old man, with a medical history of hypertension managed with calcium channel blockers, presented with missing maxillary right premolars and a maxillary horizontal deficiency. (Figure 2A). An X-ray was performed.

A vertical incision above the canine (tooth #13) was made and the flap prepared; the dermal matrix was suitably shaped and inserted into the tunnel flap. The sticky graft was then inserted inside the tunnel formed between the matrix and the bone plate.

**Figure 3:**
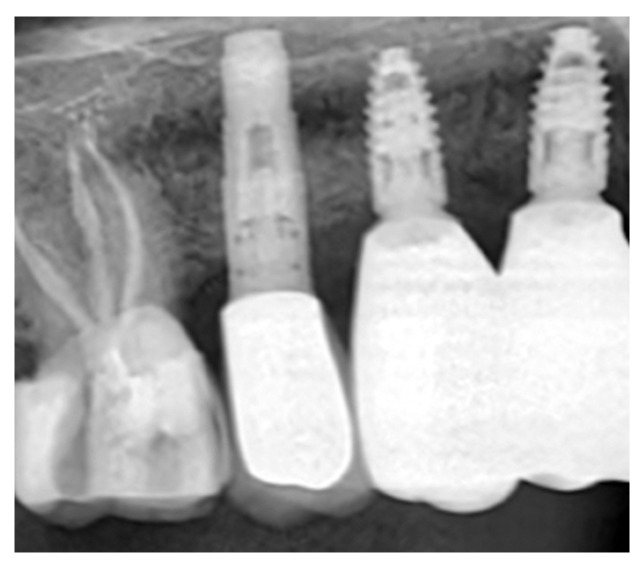
Rx at the 3-year follow-up showing stable bone integration of the implants and preservation of marginal bone levels.

The vertical incision was then closed with simple interrupted sutures. (Figure 2B) Six months after the surgery, a CBCT scan was performed. Digital planning was performed using implant software, and two implants (BLT 3.3 mm × 8 mm, Straumann) were placed with insertion torques of 40 N·cm and 45 N·cm, respectively (Figure 2C). A zirconia fixed partial denture was screwed after two months (Figure 2D). After three years, an X-ray was performed(Figure 3). Upon palpation and probing, the periodontal tissues were healthy and showed no signs of inflammation.

### 2.2. Case 2

**Figure 4:**
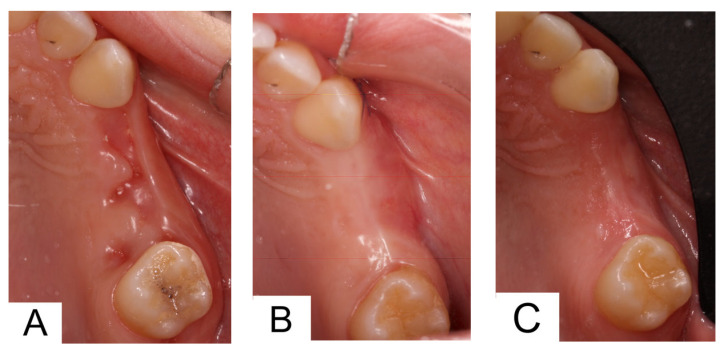
(**A**): Occlusal view after socket preservation. (**B**): Horizontal tissue augmentation after surgery. (**C**): soft tissues 4 months after tissue augmentation surgery.

A 42-year-old woman, under treatment with triptans for migraine and calcium channel blockers for arterial hypertension, presented with hopeless maxillary left premolars and first molar. Socket preservation was performed with deproteinized bone and collagen matrix. During the early stages of healing, the collagen matrix inserted to seal the graft in the socket came off. Despite socket preservation, after two months, there was evidence of buccal bone resorption (Figure 4A). Horizontal ridge augmentation was performed via a subperiosteal tunnel created through a vertical buccal incision above the canine (tooth #23). The dermal matrix was positioned, and the sticky bone was inserted. Primary closure was achieved (Figure 4B). Postoperative healing was uneventful (Figure 4C), and minimal discomfort was reported by the patient. After six months, a CBCT scan was performed, and two implants (BLT 3.3 mm × 8 mm, Straumann) were placed with insertion torques of 35 N·cm and 40 N·cm, respectively. After 2 months a zirconia fixed partial denture of two premolars were placed.

**Figure 5:**
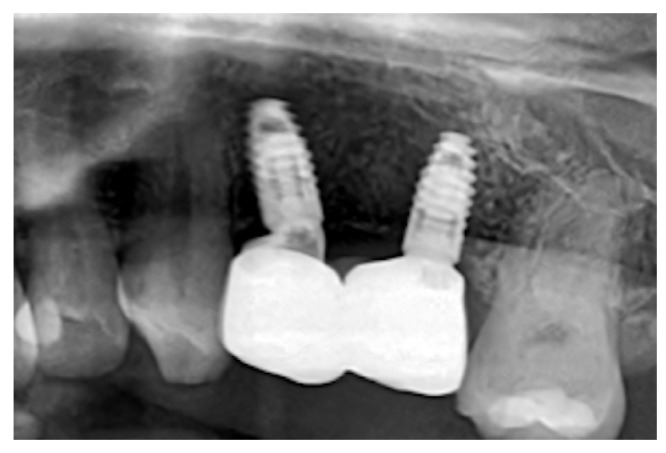
Rx at the 3-year follow-up showing complete bone integration, stable marginal bone levels, and healthy peri-implant tissue contours.

There were no signs of peri-implant inflammation upon follow-up examination and palpation after three years. Additionally, a follow-up X-ray was performed. (Figure 5).

### 2.3. Parameters Assessed and Final Outcome

**Figure 6:**
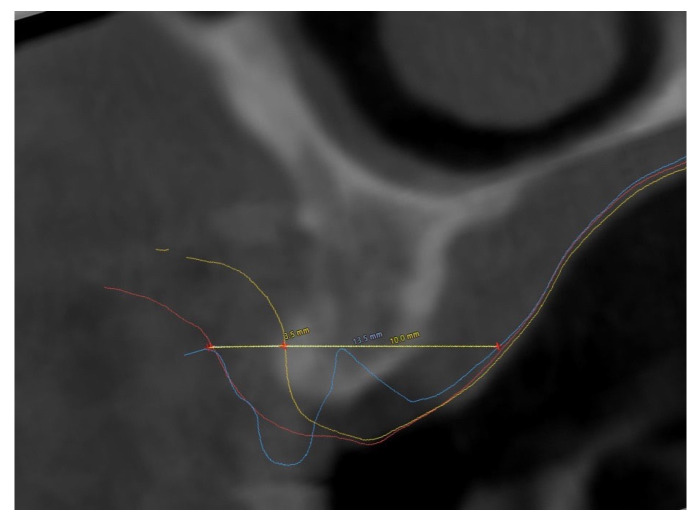
Digital comparison of alveolar ridges in Implant T0 (Yellow line), T1 (Red line), T2 (Blue line).

Clinical parameters (implant probing depth) and patient postoperative morbidity (pain score on VAS scale: 0, no pain; 10, severe pain) and analgesic consumption in the first week were recorded. The assessment of horizontal thickness increase in the alveolar ridge was observed within the implant planning software (coDiagnostiX, Dental Wings) by measuring the variation in the width of the alveolar ridge by matching three scans. It was decided not to measure it in the mouth of the patient since the probe could have compressed soft tissue and provided unreliable measurements. The scans were conducted at baseline (T0), six months after ridge augmentation (T1), and two months after implants insertion (T2). The matching was performed using corresponding pairs of regions. Cross-sectional analysis was performed at the level of the implant platform for all four implants, intersecting the deepest portion of the transmucosal pathway. Alveolar ridge thickness at this level was measured in millimeters using the ‘distance’ tool, along a line oriented parallel to the ground plane [21]. (Figure 6)

## 3. Results

This report represents a proof-of-concept presentation of two illustrative cases included within an ongoing clinical trial. Two patients were included in the study, both non-smokers and in good general health, except for controlled arterial hypertension. No relevant systemic contraindications to oral surgical procedures were present in either case. The planned intervention was carried out as initially designed, with no modifications required during treatment execution. No additional procedures were performed to augment the soft tissues. No complications were reported. No patient experienced excessive pain, swelling, bleeding, or bruising, graft explosion, flap dehiscence as well post-operative infections were observed. Patient-reported outcomes were assessed in both cases and are summarized in Table 1. These included the average postoperative pain experienced during the first week following surgery, as well as the total number of analgesic tablets consumed during that same period.

Clinical parameters at the baseline and at final follow-up where recorded as shown in Table 2

In both cases, ridge augmentation interested the maxilla. In the first case, the alveolar ridge’s horizontal thickness at the baseline (T0) was 10 mm at implant #15 and 9 mm at implant #14; from the superimposition of T1 and T2, it has been measured a thickening of 13.5 mm at implant #15 and 12.1 mm #14’s section. The gained horizontal tissue thickness has been calculated to be, respectively, 3.5 mm and 3.1 mm. The mean increased thickness at the implants site was 3,3 mm. In the second case, the initial ridge thickness was measured to be 11.1 mm for #24 and 11.3 mm #25. At T1 and T2 in the section where implant #24 was placed, the alveolar width was measured to be 13.1 mm, in the implant #25 was measured to be 13.5 mm. The mean increase in the horizontal bone thickness was, respectively, 2 mm and 2.2 mm. The mean increased thickness at the implants site was 2,1 mm. Also, in both cases, no variations in horizontal thickness were observed comparing the post-tissue augmentation scan six months after surgery and the prosthetic digital impression taken two months after implant placement. The following prostheses were screw-retained monolithic zirconia.

## 4. Discussion

The complications of horizontal augmentation techniques have been widely discussed in the international literature [22]. Soft tissue management surely represents the key to the success of regenerations, achieving primary intention closure and passivation of the flap generally considered the crucial steps for all the regenerative techniques involving a crestal incision. The tunnel approach through a vertical incision allows the operator to avoid difficult flap management and favoring an easily achievable closure. The resulting advantages have been previously described in [2] and are mainly represented by the possibility to access the bone without damaging soft tissues, promoting faster and effective healing effectively preventing the onset of complications such as bleeding, swelling, and infection.

Although flap detachment in the tunnel technique often occurred easily by a single incision, in cases with complex and irregular bone defects, detachment can be very challenging [23,24], thus surely represented a limitation to its use, like also the need to perform a second surgery for implant insertion with a lengthening the overall treatment time [25]. The materials used in this case reports were dermal matrix, deproteinized bovine bone, and hyaluronic acid. At this regard, there are techniques described in the literature that use dermal matrices for soft tissue augmentation and also as protective membranes for a graft [26,27]. The benefit extends beyond safeguarding the bone graft against soft tissue invagination, as it also results in an added enhancement of soft tissue [28,29].

The use of hyaluronic acid in addition to the xenogeneic bone allows for a sticky bone that is easy to manipulate and insert between the recipient bed and the dermal matrix [30,31]. The use of hyaluronic acid in regenerative surgery have been discussed in medicine, and its use both in dentistry and maxillofacial regenerative is rapidly expanding [32]. Many studies have shown that the hyaluronic acid has a significant positive effect on the process of osteogenesis, playing a vital role in stimulating bone growth by facilitating the distribution and densification of newly formed bone while altering scaffold morphology and promoting mineralization. Moreover, when used in combination with other biomaterials, hyaluronic acid produces biomimetic surfaces, thus promoting mesenchymal adhesion, proliferation, differentiation, and migration [31]. These processes are regulated by the transmembrane receptor CD44, which interacts with hyaluronic acid. CD44 pathways are crucial for the early inflammatory response, cell migration, and granulation tissue formation, enhancing the beneficial effects of hyaluronic acid [32]. Hyaluronic acid-based derivatives are advantageous in scaffold formation due to their ability to exert their antimicrobial properties on pathogens such as staphylococcus, streptococcus, Pseudomonas aeruginosa, entero-coccus, and S. mutants. Additionally, modifications in hyaluronic acid structure can alter its biochemistry and properties, including viscoelasticity, enhancing its potential as a biomaterial [31,32,33].

As for the dimensional changes in the edentulous ridge, they were evaluated by superimposing preoperative, postoperative, and pre-prosthetic intraoral scans. This kind of digital analysis allowed for an objective evaluation of alveolar thickness variation without subjecting the patients to further CBCTs [20]. However, it was not possible to carry out a more thorough analysis of the volumes of hard and soft tissues separately.

Our findings are in agreement with previously published clinical studies on horizontal ridge augmentation, in which bone grafts and non-stabilized dermal matrices were used through conventional approaches involving flap elevation and crestal incisions [34,35]. It should be noted that no biopsies or histomorphometric analyses were performed in this study. Consequently, the outcomes described can be interpreted only in terms of tissue thickness increase rather than true bone regeneration. Although the clinical and radiographic findings suggest favorable dimensional changes in the alveolar ridge, the absence of histological confirmation prevents definitive conclusions regarding the quality and nature of the newly formed tissue. Future studies incorporating histologic and histomorphometric evaluation are therefore needed to validate the regenerative potential of this approach [36,37].

The clinical findings presented in this two-case clinical report highlight the potential of combining xenogeneic bone, hyaluronic acid, and a dermal matrix within a tunnel approach for horizontal ridge augmentation. Although this method offers encouraging clinical and radiographic results, several aspects remain to be further explored to optimize both biological and technical outcomes. Future research should not only focus on the development and characterization of biomaterial carriers capable of providing simultaneous clot stabilization and barrier function, thus reducing the need for traditional membranes in regenerative surgery, but also on the continuation of this single-arm clinical trial to strengthen the current findings. Furthermore, a subsequent randomized study including a control group is warranted to validate the observed clinical outcomes and assess the comparative effectiveness of this approach. Conventional barrier membranes, although effective in GBR are associated with several limitations, such as the need for flap advancement, risk of exposure, bacterial contamination, and prolonged healing periods. The identification of alternative materials that can perform both mechanical and biological functions represents an important step toward more predictable and minimally invasive regeneration strategies. Among the most promising candidates are hydrogel-based or polymeric carriers enriched with bioactive molecules, such as collagen derivatives, or platelet concentrates, that can mimic the natural extracellular matrix. These materials have the ability to stabilize the blood clot in the early healing phase, preserving the space necessary for tissue ingrowth and acting as a temporary protective layer against soft tissue collapse or graft migration. When applied in combination with particulate xenogeneic bone, such carriers could form a cohesive and moldable compound, maintaining their shape within the tunnel and supporting angiogenesis. The dual function of these next-generation carriers, mechanical stability and biological activation, could eliminate the need for a separate barrier membrane, simplifying the surgical workflow and reducing postoperative morbidity. In particular, cross-linked hyaluronic acid matrices or collagen-hydrogel composites could be investigated as natural “bio-barriers” capable of maintaining the regenerative space while enhancing cellular migration and differentiation. These materials may also serve as controlled-release systems for osteoinductive growth factors or stem cell-derived vesicles, further enhancing bone regeneration potential.

Moreover, future clinical investigations should evaluate long-term volumetric stability and tissue quality through high-resolution digital analysis and advanced imaging, such as intraoral scanning superimposition and 3D morphometric evaluation. This would allow for a more comprehensive understanding of the interplay between hard and soft tissue remodeling in tunnel-guided regenerative procedures.

Another important direction involves studying the biomechanical behavior and degradation kinetics of these clot-stabilizing biomaterials, particularly their interaction with host tissues under different vascular and inflammatory conditions [38,39]. Understanding how such carriers integrate, resorb, and modulate the healing cascade will be crucial for validating their safety and predictability in clinical practice. The integration of bioactive clot stabilizers with inherent barrier properties represents a promising frontier in bone regeneration. By eliminating the need for traditional membranes, this approach could simplify surgical procedures, preserve flap integrity, and reduce patient morbidity while maintaining or even improving regenerative outcomes. Further multicenter clinical trials and translational studies will be necessary to confirm these preliminary hypotheses and define standardized protocols for the next generation of minimally invasive regenerative surgeries.

## 5. Conclusions

Within the limitations of this case series, the results suggest that the described tunneling technique may be a viable option for horizontal alveolar ridge augmentation aimed at prosthetically driven implant placement. To comprehensively assess the effectiveness of this approach, combining a hyaluronate-enriched bone graft and an acellular dermal matrix, in promoting horizontal hard- and soft-tissue regeneration while minimizing complications and patient discomfort, further investigations are required. Future studies should include histological analyses, larger sample sizes, and controlled comparative trials with longer follow-up periods to validate and expand upon these preliminary findings.

## Figures and Tables

**Table 1:** Patient-reported outcomes.

Parameters	Case 1	Case 2
**Patient-reported outcomes**		
**Postoperative pain score (average pain score in first week after su** **r** **gery)**	0.45	0.30
**Analgesic consumption (number of tablets consumed in first week)**	5	4

**Table 2:** Recorded to the nearest millimeter (mm) at the mid-buccal surface of the involved teeth with a calibrated periodontal probe (University of North Carolina [UNC]–15 probe, Hu-Friedy, Chicago, IL).

	Case 1		Case 2	
Parameters	Baseline.	3 Years Follow-Up	Baseline.	3 Years Follow-Up
** 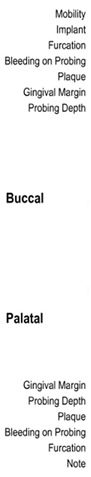 **	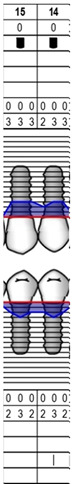	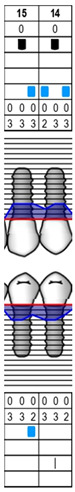	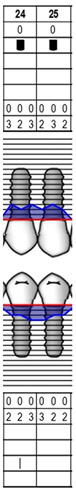	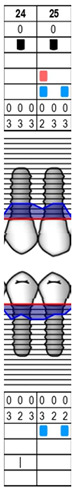

## Data Availability

The original contributions presented in this study are included in the article. Further inquiries can be directed to the corresponding authors.
